# Further psychometric validation of the BODY-Q: ability to detect change following bariatric surgery weight gain and loss

**DOI:** 10.1186/s12955-017-0802-x

**Published:** 2017-11-25

**Authors:** Anne F. Klassen, Stefan J. Cano, Manraj Kaur, Trisia Breitkopf, Andrea L. Pusic

**Affiliations:** 1 McMaster University, 3N27, 1280 Main Street W, Hamilton, ON L8N 3Z5 Canada; 2 Modus Outcomes, Letchworth Garden City, Letchworth, UK; 3 School of Rehabilitation Sciences, Institute of Applied Health Sciences, Room 308, 1400 Main Street West, Hamilton, ON L8S 1C7 Canada; 4 Memorial Sloan-Kettering Cancer Center, 1275 York Ave, New York, NY 10065 USA

**Keywords:** Patient-reported outcomes, Body-Q, Responsiveness, Clinical change, Bariatric surgery, Obesity, Quality of life, Appearance, Satisfaction

## Abstract

**Background:**

Recent systematic reviews have identified that current patient-reported outcome instruments have content limitations when used to measure change following bariatric surgery. The aim of this study was to measure change after bariatric surgery using the BODY-Q, a PRO instrument designed for weight loss and body contouring.

**Methods:**

The BODY-Q is composed of 18 independently functioning scales and an obesity-specific symptom checklist that measure appearance, health-related quality of life (HR-QOL) and experience of health-care. The sample for this study included patients who were exploring or seeking bariatric surgery in Hamilton (Canada) at the time of the BODY-Q field-test study and who agreed to further contact from the research team. These patients were invited to complete 12 BODY-Q scales and the symptom checklist between 7 June 2016 and 29 November 2016. Data were collected online (REDCap) and via postal surveys. Clinical change was measured using paired t-tests with effect sizes and standardized response means.

**Results:**

The survey was completed by 58 of 89 (65%) pre-bariatric participants from the original BODY-Q field-test sample. The non-participants did not differ from participants in terms of age, gender, ethnicity, BMI or initial BODY-Q scale scores. Participants who had undergone bariatric surgery had a mean BMI of 49 (SD = 7) at time 1 and 35 (SD = 7) at time 2. Time since bariatric surgery was on average 2 years (SD = 0.5) (range 0.4 to 3 years). Percentage total weight loss ranged from 12 to 51 (mean 31, SD = 9). The difference in the proportion of patients to report an obesity-specific symptom on the BODY-Q checklist was significantly lower at follow-up for 5 of 10 symptoms. Participants improved on BODY-Q scales measuring appearance (of abdomen, back, body, buttocks, hips/outer thighs, inner thigh), body image and physical function (*p* < 0.001 on paired t-tests) and social function (*p* = 0.002 on paired t-test). These changes were associated with moderate to large effect sizes (0.60 to 2.29) and standardized response means (0.47 to 1.35).

**Conclusions:**

The BODY-Q provides a set of independently functioning scales that measure issues important to patients who undergo weight loss. BODY-Q scales were responsive to measuring clinical change associated with weight loss 2 years after bariatric surgery.

## Background

Evidenced-based patient-reported outcome (PRO) data is needed for bariatric surgery [1–4]. PRO instruments measure outcomes that matter to patients (e.g., symptoms, health-related quality of life (HR-QOL)), by asking them directly [5]. Such instruments are being used worldwide to inform patient care, as quality metrics, in audit studies and in comparative effectiveness research [6–9]. Some countries (e.g., United Kingdom, Netherlands, Sweden) use PRO instruments at a national level to compare providers or in clinical registries [8, 9].

Outcome measures should be fit for purpose, i.e., capture the concepts of interest (e.g., physical function) in the context of use (e.g., patients attending a bariatric surgery clinic) [5]. A number of recent systematic reviews have pointed out limitations in the content of current PRO instruments measuring HR-QOL in bariatric surgery research [2–4]. For example, the most common PRO instrument used in bariatric research is the generic Short Form-36 (SF-36) http://www.qualitymetric.com. This instrument has been critiqued for failing to measure a range of concepts important to patients undergoing weight loss (e.g., sexual function, self-esteem, appearance) [10]. The most common obesity-specific tools used in bariatric research include the Impact of Weight on Quality of Life-Lite (IWQOL-Lite) [11, 12], and Moorehead-Ardelt Quality of Life Questionnaire (MAQOL) [13]. These instruments measure a range of obesity-specific concepts. The IWQOL-Lite, for example, measures physical function, self-esteem, sexual life, public distress and work [12], while the MAQOL has a total of 6 items covering self-esteem, physical, social, work, sexual and eating behavior [13]. A limitation of these obesity-specific instruments is that their measurement model (classical test theory; CTT) lacks evidence that the summed scores provide meaningful measurement [14, 15]. For example, in the CTT approach, it is considered legitimate to provide a total score for a PRO instrument that adds up scores for scales (IWQOL-Lite) or items (MAQOL). This approach to measurement is not helpful in clinical trials as it can mask effects of treatment [14, 15], for example when patients who undergo weight loss improve on some scales or items (e.g., physical function as they lose weight) and not others (e.g., body image because of the development of excess hanging skin).

The BODY-Q [16] represents a new generation PRO instrument that was developed using a modern psychometric approach called Rasch Measurement Theory (RMT) [17]. In RMT, scales that compose a PRO instrument are each designed to measure and score a unidimensional construct (no total score). In scale development, data that meet the requirement of the Rasch model provide interval-level measurement [15]. When a scale has high content validity and is targeted to measure a concept as experienced by a sample, accurate tracking of clinical change can be achieved [15].

The aim of the present study was to measure clinical change for 13 BODY-Q scales/checklist that measure appearance (of upper arms, abdomen, back, body, buttocks, inner thighs and hips/outer thighs) and HR-QOL (body image, obesity-specific symptoms and psychological, social, sexual and physical health) following bariatric surgery for participants from the BODY-Q field-test study who were recruited from the St Joseph’s Healthcare Hamilton bariatric program in Canada.

## Methods

### Body-Q

We previously described the development of the BODY-Q [16]. In phase 1, following a literature review [18] and 63 patient interviews [19], a conceptual framework and set of scales were developed to measure concepts that matter to weight loss and body contouring patients. The scales were further refined through 22 patient interviews and input from 9 clinical experts [19]. In phase 2, the scales evidenced reliability, validity, and responsiveness in an international (Canada, United States, and United Kingdom) sample of 403 pre- and post-weight loss and 331 pre- and post-body contouring surgery patients [16]. The BODY-Q is composed of 18 independently functioning scales that measure three domains (appearance (*n* = 9), HR-QOL (*n* = 5) and experience of health-care (*n* = 4)). In addition, there is a 10-item obesity-specific symptom checklist that is part of the HR-QOL domain. The follow-up survey included 12 BODY-Q scales and the obesity-specific checklist (see Table 1), alongside demographic and weight-specific (current weight and satisfaction with current weight) questions. The experience of health-care scales were excluded due to potential recall bias given the length of time elapsed since bariatric surgery for many of the participants. Also excluded were the appearance scales measuring excess skin as these were not completed by pre-bariatric patients at time 1, and body contouring scars as these were not applicable to most participants.

**Table 1:** BODY-Q scales/checklist included in follow-up study

Name of scale	Items	Example item	Response options
Body	10	How your body looks in summer clothes (e.g., shorts, t-shirts)?	dissatisfied/ satisfied
Abdomen	7	How your abdomen looks in a swimsuit?	dissatisfied/ satisfied
Arms	7	How your upper arms look when you lift them up?	dissatisfied/ satisfied
Back	4	How your back looks when you are naked?	dissatisfied/ satisfied
Buttocks	5	The size of your buttocks?	dissatisfied/ satisfied
Hips & Outer Thighs	5	The shape of your hips and outer thighs?	dissatisfied/ satisfied
Inner Thighs	4	How the skin on your inner thighs looks?	dissatisfied/ satisfied
Body Image	7	My body is not perfect but I like it.	disagree/ agree
Physical	7	Walking up or down stairs?	all the time/ never
Psychological	10	I am emotionally strong.	disagree/ agree
Sexual	5	I am comfortable having the lights on during sex.	disagree/ agree
Social	10	People listen to what I have to say.	disagree/ agree
Obesity-specific symptoms	10	Short of breath with mild exercise?	all the time/ never

### Sample

In the field-test study, which took place between November 2013 and July 2014, participants were asked if they would be willing to complete additional follow-up surveys. Of the 354 participants recruited from the St Joseph’s Healthcare Hamilton bariatric program, 107 were exploring or seeking bariatric surgery at that time. Of these, 13 did not provide permission for follow-up surveys, 1 was deceased and contact details were missing for 4. For the 89 remaining subjects, the survey was sent between 7 June 2016 and 29 November 2016, with all follow-up reminders sent by 29 November 2016.

### Recruitment method

Research subjects with an email address were sent a link to complete the BODY-Q in Research Electronic Data Capture (REDCap), a secure web-based application [20]. Subjects without an email address, and subjects whose email address was no longer valid (email bounced back) were contacted by phone. Subjects for whom we did not have an email or phone, and anyone we could not reach by email or phone, were sent the BODY-Q in the mail. Up to 2 e-mailed reminders, spaced by 1 week, and/or 2 postal reminders spaced by 3 weeks, were sent to non-respondents. Subjects received up to 2 phone call reminders as necessary.

### Statistical analysis

To identify respondent bias, non-respondents to the follow-up study were compared with respondents to the baseline survey on the following variables: age (continuous), gender (male vs. female), ethnicity/race (white vs. other), BMI (continuous) and initial BODY-Q scale scores. BODY-Q scores range from 0 (worse) to 100 (best) scores based on Rasch logits developed in the field-test sample. Analysis included Chi-square tests to examine differences in categorical variables, and t-tests or the equivalent nonparametric test depending on the normality of the distribution of the scores.

For the obesity-specific symptom checklist, the difference in scores from time 1 to time 2 for each symptom was computed. In addition, responses were rescored to form dichotomous *yes* (sometimes/often/always bothered) versus *no* (never bothered) variables for each of the 10 symptoms. Chi-square tests were used to examine the significance of change in the proportion of participants to report a symptom before and after bariatric surgery.

Clinical change was measured by computing paired t-tests, or the nonparametric equivalent for data without a normal distribution, and computing the effect sizes as described by Kazis et al. [21] and standardized response means [22]. The magnitude of the change was interpreted using Cohen’s arbitrary criteria (i.e., small, 0.20; moderate, 0.50; and large, 0.80) [23]. Pearson correlations were computed between the change scores (i.e., mean time 1 – mean time 2) for BODY-Q scales and percentage total weight gain/loss (%TWL) calculated as follows: (((weight at the time of bariatric surgery – current weight)/weight at the time of bariatric surgery) * 100)).

Data were analysed using SPSS Version 23 [IBM SPSS Statistics, Version 23, IBM Corp]. All statistical tests, *p*-values <0.05 were considered statistically significant.

## Results

The survey was completed by 58 (response rate 65%) of the 89 participants who were sent an invitation to complete the follow-up survey. The 58 participants did not differ from the 49 participants non-participants who provided data at baseline in terms of age, gender, ethnicity, BMI, or any of the BODY-Q scores completed by the sample at time 1.

Of the 58 participants, 4 had not undergone bariatric surgery at follow-up and were excluded from the subsequent analyses. Of the 54 remaining participants, age ranged from 27 to 71 years (mean = 48, SD = 12), 42 (77.8%) were female, and 44 (81.5%) were Caucasian. The mean time since bariatric surgery was 2 years (SD = 0.5; from 0.4 to 3 years). Baseline BMI was 50 (SD = 7), and at follow-up was 35 (SD = 7). All participants lost weight: mean %TWL was 31 (SD = 9; from 12 to 51). Dissatisfaction with weight was reported by 51 (95%) participants before bariatric surgery and 20 (37%) participants at follow-up. Body contouring to remove excess skin had been obtained by 3 (6%) participants, while 40 (74%) participants indicated that they needed body contouring to remove excess skin from one or more areas of their body.

### Change in obesity-specific symptoms

Table 2 shows the number of participants for each possible change in score (deteriorate, stay the same, improve) for each obesity-specific symptom. The item with the most change was “Short of breath with mild exercise?” Here, 36 participants improved by at least one response option. The item with the highest number of participants (*N* = 10) to report a worse score at follow-up was “Feeling off balance?”

**Table 2:** Number of participants to deteriorate, stay the same or improve from before to after bariatric surgery for each obesity-specific symptom

Obesity-specific symptoms	Deteriorate			Stay same	Improve		
	-3	−2	−1	0	+1	+2	+3
1. Feeling tired during the day	0	1	6	20	18	2	2
2. Back pain	0	2	4	25	12	5	0
3. Joint pain	0	1	6	23	7	11	1
4. Leg pain or discomfort	0	2	3	18	14	9	3
5. Feeling off balance	0	1	9	17	19	3	0
6. Feeling weak	0	0	7	23	15	4	0
7. Short of breath with mild exercise	0	0	1	12	22	7	7
8. Swollen feet	0	0	7	18	15	8	1
9. Skin rash or infection	0	3	4	30	6	5	1
10. Too much perspiration	0	1	3	27	15	1	2

*Note*: Missing data ranged up to 6 per item

Table 3 shows the change in the proportion of the sample to report a symptom (dichotomized into never vs sometimes/often/all the time) before and after bariatric surgery for each obesity-specific symptom. The 3 most common symptoms (items 1–3) prior to bariatric surgery were also highly endorsed at follow-up. The difference in the proportion of participants to report a symptom was significantly lower at follow-up for 5/10 symptoms.

**Table 3:** Number (%) of participants to report an obesity-specific symptom (yes/no) before and after bariatric surgery, and Chi-square -value for test of significance

Symptom	Time 1		Time 2		*p*-value
	Yes	No	Yes	No	
1. Feeling tired during the day	47 (95.9)	2 (4.1)	44 (89.8)	5 (10.2)	1.000
2. Back pain	44 (91.7)	4 (8.3)	41 (85.4)	7 (14.6)	0.096
3. Joint pain	44 (89.8)	5 (10.2)	42 (85.7)	7 (14.3)	0.143
4. Leg pain or discomfort	41 (83.7)	8 (16.3)	30 (61.2)	19 (38.8)	0.004
5. Feeling off balance	34 (69.4)	15 (30.6)	20 (40.8)	29 (59.2)	0.597
6. Feeling weak	35 (71.4)	14 (28.6)	25 (51.0)	24 (49.0)	0.012
7. Short of breath with mild exercise	43 (87.8)	6 (12.2)	22 (44.9)	27 (55.1)	0.027
8. Swollen feet	32 (65.3)	17 (34.7)	20 (40.8)	29 (59.2)	0.126
9. Skin rash or infection	24 (49.0)	25 (51.0)	18 (36.7)	31 (63.3)	0.019
10. Too much perspiration	29 (59.2)	20 (40.8)	21 (42.9)	28 (57.1)	0.001

### Change in BODY-Q scores

Table 4 shows the mean scores before and after bariatric surgery, mean difference in scores, *p*-value, effect sizes and standardized response means. Significant higher satisfaction with appearance was reported for all areas except for the Upper Arms scale. For the HR-QOL scales, a significant change was reported for Body Image, Physical and Social. These changes were associated with moderate to large effect sizes (0.60 to 2.29) and standardized response means (0.47 to 1.35).

**Table 4:** Mean scores pre- and post-bariatric surgery, p-value for paired t-test and Effect Size

	No.	Pre Mean (SD)	Post Mean (SD)	Mean difference (SD)	*p*	ES	SRM
Arms	53	25.2 (19.3)	28.8 (27.2)	3.6 (23.8)	0.272	0.19	0.15
Abdomen ^a^	53	5.8 (10.6)	25.1 (23.9)	19.3 (24.7)	<0.001	1.82	0.78
Back	51	21.1 (22.1)	48.0 (27.8)	26.9 (29.1)	<0.001	1.22	0.92
Body	53	14.7 (12.3)	42.9 (18.6)	28.3 (20.9)	<0.001	2.29	1.35
Buttocks	50	16.0 (20.0)	39.3 (24.1)	23.2 (25.5)	<0.001	1.16	0.91
Hips Outer Thighs	51	15.8 (19.9)	38.9 (24.3)	23.1 (26.0)	<0.001	1.16	0.89
Inner Thighs	51	10.5 (15.1)	27.9 (28.2)	17.4 (27.2)	<0.001	1.15	0.64
Body Image	51	13.4 (13.6)	37.2 (22.3)	23.7 (22.2)	<0.001	1.74	1.07
Physical	49	46.1 (19.7)	76.2 (21.4)	30.1 (23.2)	<0.001	1.53	1.30
Psych	50	55.5 (17.7)	58.4 (21.7)	2.9 (17.6)	0.254	0.16	0.17
Sexual	46	44.7 (21.5)	43.0 (23.5)	−1.6 (21.6)	0.615	−0.08	−0.07
Social	50	51.4 (17.0)	61.6 (19.5)	10.2 (21.9)	0.002	0.60	0.47

*ES* effect size, *SRM* standardized response mean

^a^Nonparametric test performed

### Correlation between %TWL and BODY-Q scores

More improvement in BODY-Q scores correlated with higher %TWL for the following scales: Body (*r* = 0.52, *p* < 0.001), Upper Arms (*r* = 0.31, *p* = 0.024), Back (*r* = 0.39, *p* = 0.005), Buttocks (*r* = 0.34, *p* = 0.018), Hips and Outer Thighs (*r* = 0.29, 0.044), Body Image (*r* = 0.45, *p* = 0.001) and Sexual (*r* = 0.37, *p* = 0.011).

## Discussion

The BODY-Q represents a new generation PRO instrument developed and validated using a modern psychometric approach to provide a set of unidimensional, scientifically sound scales that measure concepts of interest important to patients undergoing weight loss and/or body contouring. Our findings show that BODY-Q scales were responsive to measuring clinical change in patients who underwent bariatric surgery. The variation in effect sizes and standardized response means, from no change to moderate and large change across scales illustrates why it is important to provide separate results for each scale rather than sum to produce a total score for scales measuring different concepts.

Bariatric surgery is often pursued by people aiming to improve their physical and psychosocial HR-QOL. However, massive weight loss often leads to excessive skin, which can have a negative influence on HR-QOL [24]. The BODY-Q was specifically designed to measure outcomes important to patients over the entire patient journey starting at obesity and ending after body contouring to remove excess skin. We found that participants improved in terms of body image, social and physical function, and reported fewer obesity-specific symptoms. Our sample did not improve in terms of psychological and sexual function. These findings are in line with recent systematic reviews of intermediate and long term HR-QOL in bariatric surgery. Jumbe et al. [3] examined 11 studies focused on the long-term effectiveness of bariatric surgery on quality of life compared to non-surgical interventions. These researchers found that long-term psychosocial quality of life did not appear to improve following bariatric surgery despite significant improvements in physical quality of life over time. Similarly, Kubik et al. [25] evaluated the impact of bariatric surgery on psychological functioning based on 27 articles and concluded that bariatric surgery results in mental health gains in the short-term, but this effect was not sustained beyond two years’ post-surgery. Hachem and Brennan [4] compared bariatric surgery with alternative weight-loss interventions and between different bariatric surgical procedures based on 15 controlled trials and showed that physical function consistently improved following bariatric surgery, but that improvements in mental health and psychosocial function were mixed. Raaijmakers et al. [2] examined short and long-term effects of bariatric surgery compared with community norms based on 36 studies and found that while quality of life significantly improved after surgery, a few studies showed no significant improvement in the mental health component of quality of life. Authors from these reviews pointed out limitations in current literature (e.g., lack of consensus on the choice of PRO instruments used) and called for more research to gain a greater understanding of the effects of bariatric surgery on the health of patients.

Though our sample is small, the authors of a large cross-sectional study of 493 bariatric surgery patients from Denmark compared BODY-Q scores for 4 phases of the weight loss journey: pre-bariatric surgery, post-bariatric surgery (4/5 or 12 months post-surgery), pre-body contouring surgery, and post-body contouring surgery [26]. BODY-Q scores for appearance and HR-QOL were lowest in the pre-bariatric phase, followed by patients in the pre-body contouring phase. The Danish findings suggest that outcomes following bariatric surgery are not optimal until body-contouring to remove excess hanging skin is performed, and they call for longitudinal research to measure the full extent of HR-QOL and appearance change following weight loss and reconstructive treatments. In the present study, the majority of participants reported that they needed body contouring surgery to remove excess skin in order to complete their weight loss journey, which could account for the lack of improvement in psychological and sexual function.

This study has some limitations. The response rate was low and our sample size was small. Although we did not find any differences between the group of non-respondents and respondents on demographic, clinical and BODY-Q scores, there could still be bias in the sample of patients who completed our survey. Another limitation is that the participants were from a single bariatric surgery centre in Canada and may not represent the bariatric patients in other centres or other countries. Finally, the clinical data collected were self-report and could have errors due to participants guessing (e.g., date of bariatric surgery).

## Conclusion

Participants in our sample were at various stages in their weight loss journey, with many requiring body contouring to remove excess skin after massive weight loss. While it is clear that bariatric surgery leads to improvement in physical health, evidence-based information is still needed to show the full extent of psychosocial, sexual and body image/appearance change that follows weight loss across the entire journey.

## Abbreviations

CTT: Classical test theory
HR-QOL: Health-related quality of life
IWQOL-Lite: Impact of Weight on Quality of Life-Lite
MAQOL: Moorehead-Ardelt Qualiy of Life Questionnaire
PRO: Patient-reported outcome
RMT: Rasch Measurement Theory
REDCap: Research Electronic Data Capture
SD: Standard deviation
%TWL: Percent total weight loss
